# Molecular Characterization and Phylogenetic Relationship of Wild Type 1 Poliovirus Strains Circulating across Pakistan and Afghanistan Bordering Areas during 2010–2012

**DOI:** 10.1371/journal.pone.0107697

**Published:** 2014-09-17

**Authors:** Shahzad Shaukat, Mehar Angez, Muhammad Masroor Alam, Salmaan Sharif, Adnan Khurshid, Farzana Malik, Lubna Rehman, Syed Sohail Zahoor Zaidi

**Affiliations:** Department of Virology, National Institute of Health, Chak Shahzad, Islamabad, Pakistan; Boston College, United States of America

## Abstract

Pakistan and Afghanistan share a long uncontrolled border with extensive population movement on both sides. Wild poliovirus transmission has never been interrupted in this block due to war against terrorism, poor public health infrastructure, misconceptions about polio vaccines and inadequate immunization activities. All these issues complicate the eradication operations and reinforce the complexity of wiping out poliomyelitis from this region. This study illustrates the origins and routes of cross-border wild poliovirus type 1 (WPV1) transmission during 2010–2012 between Pakistan and Afghanistan. Sequence analyses were conducted based on complete VP1 capsid protein sequences for WPV1 study strains to determine the origin of poliovirus genetic lineages and their evolutionary relationships. Phylogenetic tree was constructed from VP1 gene sequences applying Maximum Likelihood method using Kimura 2- parameter model in MEGA program v 5.0. A total of 72 (14.3%) out of 502 wild-type 1 polioviruses were found circulating in border areas of both countries during 2010–2012. Molecular phylogenetic analysis classified these strains in to two sub-genotypes with four clusters and 18 lineages. Genetic data confirmed that the most of WPV1 lineages (12; 66.6%) were transmitted from Pakistan to Afghanistan. However, the genetic diversity was significantly reduced during 2012 as most of the lineages were completely eliminated. In conclusion, Pakistan-Afghanistan block has emerged as a single poliovirus reservoir sharing the multiple poliovirus lineages due to uncontrolled movement of people across the borders between two countries. If it is neglected, it can jeopardize the extensive global efforts done so-far to eradicate the poliovirus infection. Our data will be helpful to devise the preventive strategies for effective control of wild poliovirus transmission in this region.

## Introduction

The World Health Assembly passed a resolution in 1988 to eradicate poliomyelitis. Since then, a marvelous progress has been made in the global fight against polio [1]. The global anti-polio campaign has been successful as the number of polio cases has decreased from an estimated 350,000 cases in 1988 to 407 cases in 2013. Pakistan, Afghanistan and Nigeria are the only three countries within Eastern Mediterranean and African regions respectively where polio is still endemic [2], [3]. Pakistan and Afghanistan together are epidemiologically considered as one block and transmission of Wild Poliovirus (WPV) has never been interrupted in this region because of poor public health infrastructure, very low routine immunization and prejudices against polio vaccination [4]–[6].

Epidemiological information and genetic data analysis of poliovirus strains is used to find out the links between polio cases, to identify local reservoir and to track the transmission pathway of the poliovirus in a specific geographic location [7], [8]. Furthermore, the genetic relatedness of poliovirus that has been circulating within a population forms lineages of closely related “chains of transmission”. This relatedness of polioviruses is a strong indicator for the sensitivity of acute flaccid paralysis (AFP) surveillance system [9].

Geographically, Pakistan and Afghanistan share a long uncontrolled border (2611 kilometer) with only two officially established crossings at Chaman in Baluchistan province and at Torkhum in Khyber Pakhtunkhwa province [10]. The border areas on the Pakistani side are constituted within the Federally Administered Tribal Areas (FATA), the Khyber Pakhtunkhwa (KP) and Baluchistan provinces. On Afghanistan side, eleven provinces share border with Pakistan, from North to South, namely: Badakhshan, Nuristan, Kunar, Nangarhar, Paktia, Khost, Paktika, Zabul, Kandhar, Helmand and Nimruz [11]. Poliovirus is transmitted between two countries through population movements of different groups that include migrant population (nomadic, seasonal, economic and Afghan migrant), transit population and Pashtu speaking population outside KP and FATA [12]. These people travel for a mixed variety of reasons, influenced by social, cultural and economic factors but the economic motivations are the main factors leading Afghans to travel to Pakistan [13], [14]. Whatever may be the reason and whether momentary or permanent, the increased number of migrants may be carrier of poliovirus infection across these areas and facilitate transmission.

In our present study, we described the transmission pathways of wild poliovirus in border areas of Pakistan and Afghanistan during the years 2010–2012. The emergence of individual chains of transmission as visualized as separate genetic lineages and data of population movement was followed to reconstruct the transmission pathways of the wild type 1 poliovirus between the two countries.

## Materials and Methods

### Ethics Statement

The study concept and design was approved through the Pakistan's National Institute of Health Internal Review Board. Written informed consent was obtained from all the patient's parents/guardians.

### Study Design

This study was designed to find out the molecular epidemiology of WPV1 circulating in bordering areas of Pakistan and Afghanistan during 2010–2012. During this period, a total of 16082 and 5232 stool samples were received at WHO Regional Reference Laboratory for the Global Polio Eradication Initiative, department of virology, National Institute of Health, Islamabad, collected from Pakistan and Afghanistan respectively.

### Virus Isolation and Characterization

All samples were processed for poliovirus isolation and identification using standard operating procedures as described previously [15]. Briefly, these samples were inoculated on L20B (murine fibroblast cells genetically engineered to express the human poliovirus receptor) [16], [17] and RD (human rhabdomyosarcoma) cell lines [18], [19] for poliovirus isolation as per recommendation of the World Health Organization's polio laboratory manual [20]. Positive isolates were differentiated in to Sabin like or non-Sabin like poliovirus through Real time reverse transcription PCR based intra type differentiation (ITD) assays [21]. All wild poliovirus type 1 strains were subjected to complete viral protein 1 (VP1) gene amplification in one-step RT-PCR assay using the Y7 forward (5′-GGT TTT GTG TCA GCG TGT AAT GA-3′) and Q8 reverse (5′-AAG AGG TCT CTA TTC CAC AT-3′) primers. The amplicons were purified and cycle sequencing reactions were performed using the ABI Prism BigDye Terminator v.3.1 kit (PE Applied-Biosystems, Foster City, CA, USA) with 25 cycles at 94°C for 20 seconds, 42°C for 15 seconds and 60°C for 4 minutes. The samples were sequenced in an automated ABI 3100 genetic analyzer (Applied Biosystems, Foster City, CA, USA).

A total of 72 cases from Pakistan (n = 39) and Afghanistan (n = 33) were selected which fulfill the inclusion criteria of the study i.e. date of paralysis onset during January 2010 to December 2012, history of residence of patient in the bordering areas/districts and isolation of WPV1 from stool sample.

The bordering area between Pakistan and Afghanistan was broadly divided into two regions. Region-1 includes the southern transmission zone comprising Quetta block (Quetta, Pishin, Killa Abdullah) in Pakistan and bordering south Afghanistan (Kandhar, Helmand) while region-2 includes the northern transmission zone constituting the FATA region (Bajour, Mohmand, Khyber, Orakzai, Kurram, North Waziristan and South Waziristan), some part of KP in Pakistan and bordering east Afghanistan (Nangarhar, Paktia, Kapisa, Paktika). The key identifiers of provinces and districts are presented in supplementary figure (Figure S1).

### Phylogenetic analyses

The VP1 nucleotide sequences from 72 selected WPV1 strains were used to generate a dataset for phylogenetic analysis. Sequence data was analyzed using Sequencher software (Gene Codes v 4.5). Multiple sequence alignment was performed using ClustalW [22]. The distance matrix was calculated using Kimura-2 parameter [23] and Phylogenetic tree was constructed by Maximum Likelihood method using Molecular Evolutionary Genetic Analysis (MEGA v 5.0) software [24]. Sub-genotypes and clusters were defined based on the criteria as group of related strains with ≥90% and ≥95% nucleotide identity in a 906 nucleotide long sequence of complete VP1 gene fall in a same sub-genotype and cluster respectively. To compile a phylogenetic tree, distances of 1000 bootstrapped data sets were calculated by use of the Kimura-2 parameter model for evolutionary rate.

The sequence data described in this study have been submitted to Genbank data library under accession numbers KJ460261–KJ460332.

## Results

### Geographic distribution

Pakistan reported 55WPV1 cases in 2012 as compared to 120 and 196 cases reported in 2010 and 2011 respectively. In 2011, Pakistan reported the highest incidence of WPV1 with 196 cases since 2000. Similarly, Afghanistan reported a total of 17, 80 and 37 confirmed wild poliovirus type 1 cases in 2010, 2011 and 2012 respectively.

### Phylogenetic analyses

**Figure 1:**
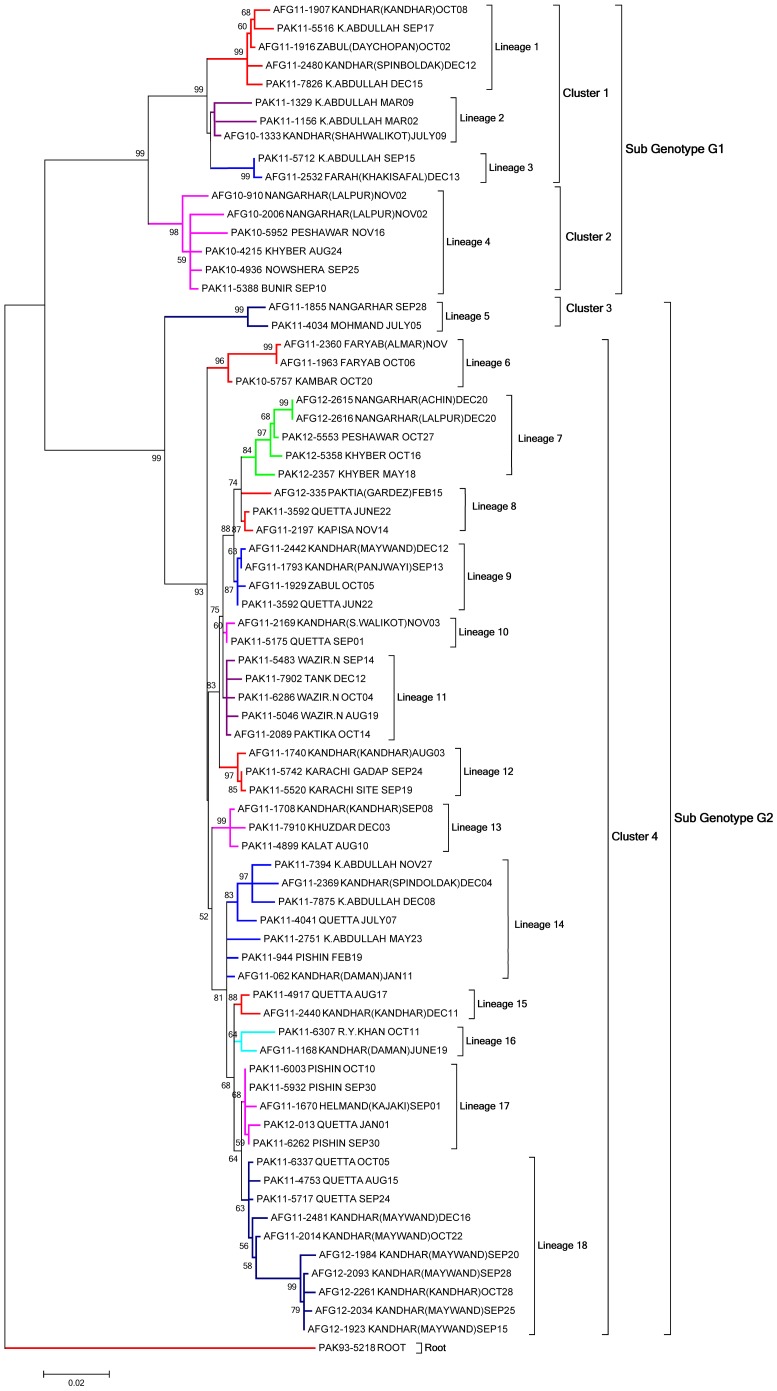
Phylogenetic analyses of Wild poliovirus type 1 isolates. The phylogenetic tree was constructed on the basis of the complete VP1 region nucleotide sequences by the Maximum Likelihood with MEGA (version 5) software, and the bootstrap values indicated at the branch nodes were evaluated using 1,000 replicates. Only values of over 50% were shown. Visualization of 18 lineages (L1–L18) within two sub-genotypes (G1 and G2) and four clusters (C1–C4) were distinguished by colored branches. Each strain is indicated by country code followed by year of isolation, strain ID, region of isolation and the onset of paralysis. The WPV1 strain (PAK93-5218) isolated in 1993 was used as root.

The phylogenetic analyses of 72 selected AFP cases (Table 1) from border areas of both countries are summarized in an evolutionary tree (Figure 1). From the combined evolutionary analysis, geographical information and clinical history like date of paralysis onset, we traced the transmission patterns of wild poliovirus type 1 lineages circulating between Pakistan and Afghanistan borders during 2010–2012.

**Table 1:** Wild Poliovirus type 1 (WPV1) distribution among Acute Flaccid Paralysis cases from Pakistan and Afghanistan during 2010–2012.

	Pakistan			Afghanistan		
Year	No. of AFP Cases	Confirmed WPV1	WPV1 in Bordering Area	No. of AFP Cases	Confirmed WPV1	WPV1 in Bordering Area
**2010**	5382	120	4	1572	17	3
**2011**	5662	196	31	1831	80	22
**2012**	5038	55	4	1829	37	8
Total	16082	371 (2.3%)	39 (10.5%)	5232	134 (2.6%)	33 (24.6%)

In Pakistan, annual non-polio AFP rate (per 100,000 children aged <15 years) was 6.9, 7.2 and 6.3 in 2010, 2011 and 2012 respectively. In Afghanistan, this rate was 9.2, 10.5 and 9.5 in 2010, 2011 and 2012 respectively.

On the basis of our evolutionary relationship analyses, poliovirus strains were categorized in to two major sub-genotypes; G1 and G2. The mean pairwise nucleotide sequence identities within G1 and G2 sub-genotypes was 96.4% (94.3–99.4%) and 97.2% (92.9–100%) respectively. These sub-genotypes were further sub divided in to four clusters (C1 to C4) based on pairwise nucleotide sequences. The divergence observed among the strains between different clusters within same sub-genotype was at least 4.8% (and up to 6.3%). Similarly, 18 independent lineages (L1–L18) were identified circulating among the bordering areas of both countries. Each lineage within a cluster was distinguished from other by at least 0.6% (and up to 3.8%) nucleotide divergence.

### Genetic analysis of sub-genotype G1

**Figure 2:**
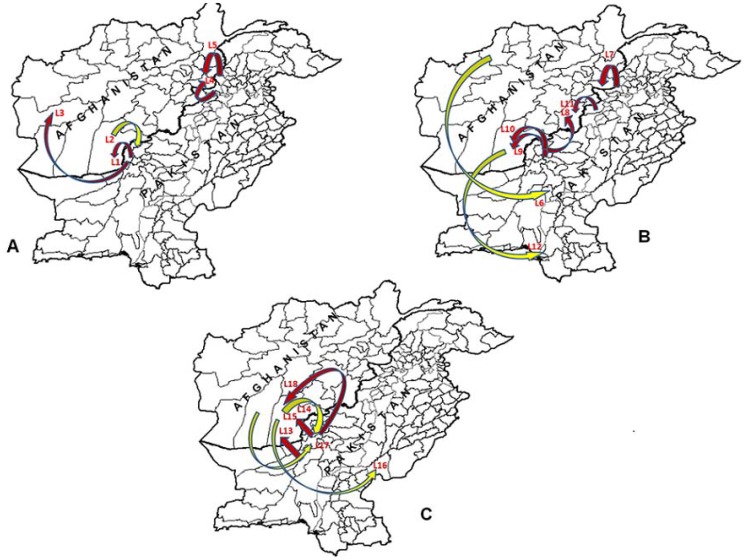
Geographic distribution of patients with clinical cases of polio associated with isolates of wild type 1 poliovirus (indicated by colored arrows) from 2010–2012 in border areas of Pakistan and Afghanistan. A) Transmission Pattern in sub-genotype G1 illustrates five lineages (L1–L5); B) Transmission Pattern in sub-genotype G2 illustrates seven lineages (L6–12) and C) Transmission Pattern in sub-genotype G2 illustrates six lineages (L13–18). The arrows represent the lineages or transmission pathways between both countries. Transmission pathways of Wild poliovirus type 1 from Pakistan to Afghanistan are represented by brown arrows while yellow indicates the transmission from Afghanistan to Pakistan.

During 2010–2011, 16 isolates were placed in sub-genotype G1 which includes 9 isolates from Pakistan and 7 from Afghanistan. These isolates shared 89.3% (87–90.8%) mean nucleotide identity with sub-genotype G2 isolates. G1 was further divided into two clusters; C1 and C2 (Figure 1). Cluster C1 had three lineages (L1 to L3) sharing 97% (96.5–97.5%) mean nucleotide identity with each other while cluster C2 had only one lineage (L4) with isolates having 98.6% (97.9–99.4%) mean nucleotide identity. The sequence data indicated a close genetic link between isolates of cluster C1 confined to Quetta block (Quetta, Pishin, Killa Abdullah) in Pakistan and southern regions (Kandhar and Helmand) of Afghanistan (Figure 2). Lineage L1 of cluster C1 was only found in Kandhar, Zabul and Killa Abdullah during 2011 while lineage L2 was introduced from Kandhar (Afghanistan) to Killa Abdullah (Pakistan) (Figure 2). This lineage remained in circulation during 2010 in Kandhar and later isolated in 2011 from Quetta block of Baluchistan province in Pakistan. Lineage 3 was originally found in Killa Abdullah (2010) but also isolated from Farah province of Afghanistan during 2011. The viruses belonging to lineage L4 were circulating in Khyber, Peshawar, Bunair and Nowshehra districts of KP province having 99% and 98.9% nucleotide identity. Later on, this lineage was found in AFP cases from Nangarhar (AFG-10 910 and AFG-10 2006) with 98.1% pairwise nucleotide identities to PV strains circulating in Khyber and Peshawar districts of KP province in Pakistan.

### Genetic analysis of sub-genotype G2

Sub-genotype G2 divided into two clusters C3 and C4 having one (L5) and 13 lineages (L6 to L18) respectively (Figure 1). Phylogenetic analysis confirmed that 56 WPV1 strains from Pakistan (n = 30) and Afghanistan (n = 26) were grouped in sub-genotype G2. Among these, 2 isolates belonged to cluster C3, while 54 belonged to cluster C4. A mean pairwise nucleotide sequence identities within clusters C3 and C4 was 98.9% and 97.7% (95–100%) respectively while 94.4% (92.9–95.3%) was observed between the two clusters. Furthermore, 13 lineages (L6 to L18) in cluster C4 showed at least 99.4% (up to 96.2%) nucleotide identity with each other. Infections with sub-genotype G2 were found more frequently in both countries during the three years study period i.e. 2010–2012. Cluster C3 comprised only one lineage (L5) that remained active only in 2011 and first originated in FATA (Mohmand) in July and then detected in an AFP case from eastern Afghanistan (Nangarhar) with onset of paralysis in September, 2011. This lineage could not be detected later in both countries.

Cluster C4 was found more active and widely distributed in bordering areas of both countries with 13 lineages during 2010–2012. The importation of WPV1 strains into bordering areas of Pakistan from Afghanistan appeared to follow two lineages; L14 and L17 (Figure 2). In 2011, L14 was first detected in southern Afghanistan (Kandhar) and then imported to Pishin, Quetta (Baluchistan) while L17 was introduced to Pishin (Baluchistan) from Helmand (Afghanistan). Furthermore, eight lineages (L7, L8, L9, L10, L11, L13, L15 and L18) were found to be imported in Afghanistan from Pakistan during 2010–2012 (Figure 1 and 2). The phylogenetic data confirmed the transmission of WPV1 from Quetta block to southern regions of Afghanistan and Kapisa during 2011–2012 through six different lineages (L8, L9, L10, L13 L15 and L18) (Figure 2). Lineages L8 and L18 were detected during 2011–2012 while others remained there only in 2011. Similarly, two lineages (L7 and L11) were imported in eastern parts of Afghanistan (Nangarhar and Paktia) from FATA (Khyber, North Waziristan) during 2011–2012. Three lineages (L7, L8 and L18) showed circulation during 2012 while all others disappeared in 2011.

In cluster C4, three lineages (L6, L12 and L16) were also found that were originated either from bordering areas or from non-bordering areas in Pakistan and Afghanistan (Figure 2). In 2010, only one isolate (PAK-10 5757) of lineage L6 from Kambar, Pakistan was most closely related (98.3%) to 2011 Faryab, Afghanistan isolates (AFG11-1963 and AFG11-2360). This lineage was found more distinct (2.4–3.8%) from other lineages in this cluster. Similarly, in 2011, lineages L12 and L16 were imported to Karachi (Sindh) and R.Y. Khan (Punjab) from Kandhar but no data from the intermediate isolates were available to trace the origin of these viruses.

In short, during 2010–2012, 18 distinct lineages of poliovirus were found circulating between both countries with majority of those originated in Quetta block and FATA region in Pakistan but later introduced in to southern and eastern regions of Afghanistan.

## Discussion

Pakistan and Afghanistan are among two of the three remaining “endemic” polio nations in Asia that have never stopped wild poliovirus transmission. Both countries have experienced repeated conflicts like war against terror for more than a decade leading to frequent migration of population especially through “FATA-eastern regions” and “Quetta block-southern regions” corridors. The main objective of this study was to highlight the transmission pathways of WPV1 strains through bordering area of both countries. Our findings underscore the importance of synchronized eradication efforts, strong cooperation and political commitment between Pakistan and Afghanistan.

Population movements between both countries appear to be more varied, complex and remains informal at all levels [13]. Decades of war and conflict have resulted in a closely knit network of contacts that make it easier for people to move between the two states. Nearly 2.5 million Afghans reside in Pakistan. They go back and forth to Afghanistan on a regular basis for social services, and to visit their relatives and friends [14], [25]. The frequent visits of Afghans residing in Pakistan show a two-way cross border movement facilitating the transmission of poliovirus across the countries. Our data also strengthens this argument because most of the lineages (n = 12) imported from Pakistan to Afghanistan followed the same transmission paths as utilized by the population moving across the borders [10]. Additionally, in these areas, the endemic transmission is going on because of consistent failure to access and immunize the children against poliomyelitis. Therefore, the movement of asymptomatic carriers from these regions to Afghanistan has resulted in exportation of poliovirus [26].

Genetic characterization of wild type 1 polioviruses can help to confirm the sources of virus as well as to establish links between poliovirus cases and outbreaks [27], [28]. In this study, two sub-genotypes (G1 and G2) of WPV1 were found co-circulating during 2010–2012. Genomic sequencing data for 2011 showed the emergence of many genetic lineages in southern transmission zone (bordering south Afghanistan and Quetta block) as well as in the northern transmission zone (FATA, some parts of KP and bordering areas of east Afghanistan). The increased genetic lineages clearly indicates the immunity gaps in these regions due to consistently compromised quality of immunization campaigns coupled with very low routine EPI coverage, precarious security situation, managerial problems and devolution of health authority in 2011 from federal to provincial government [2], [29]–[32].

On the other hand, genetic data shows a reduction in genetic transmission lineages in 2012 as only three lineages (L7, L17 and L18) were detected as compared to 2010–2011. Interestingly, two of the remaining lineages (L17 and L18) remained in circulation during 2012 but confined to southern transmission zone only. Similarly, third lineage (L7) appeared in Khyber during 2012 and later isolated in Nangarhar with increased genetic diversity. This may indicate the gaps in surveillance activities due to insecurity and inaccessibility in these areas.

Furthermore, majority of the lineages analyzed in this study were found circulating especially in the southern transmission zone. During 2012, only two lineages were found to continue in this region while 82% of the lineages were completely eliminated. The decreased genetic diversity and the improved immunization activities in this region indicate that the polio eradication target is achievable. However, improved and intensified progress is needed in the remaining pockets of transmission especially in the northern transmission zone like the Federally Administered Tribal Areas, some part of Khyber Pakhtunkhwa and bordering east Afghanistan [33], [34].

In conclusion, Pakistan-Afghanistan block has emerged as a single poliovirus reservoir sharing different poliovirus lineages. More lineages are seen to be imported from Pakistan to Afghanistan. Continuous back and forth movement of people and poliovirus infection with them requires close harmonization, coordination and team work between the two countries to control this cross-border transmission. There is dire need to develop synchronize policies to improve the immunization activities in both countries. Furthermore, the molecular epidemiological data available from this study will help the health authorities of both countries to devise strategies for poliovirus transmission control and to improve the immunity in these areas. The value of this work is regularly reaffirmed by national and WHO epidemiologists.

## Supporting Information

Figure S1 **Identifiers of Key provinces and districts in Pakistan and Afghanistan.**
(TIF)
