# Supplementary figures and images for: Molecular Characterization and Phylogenetic Relationship of Wild Type 1 Poliovirus Strains Circulating across Pakistan and Afghanistan Bordering Areas during 2010–2012
Source: PLoS One. 2014 Sep 17;9(9):e107697. doi: 10.1371/journal.pone.0107697 (PMC4168008; doi:10.1371/journal.pone.0107697)

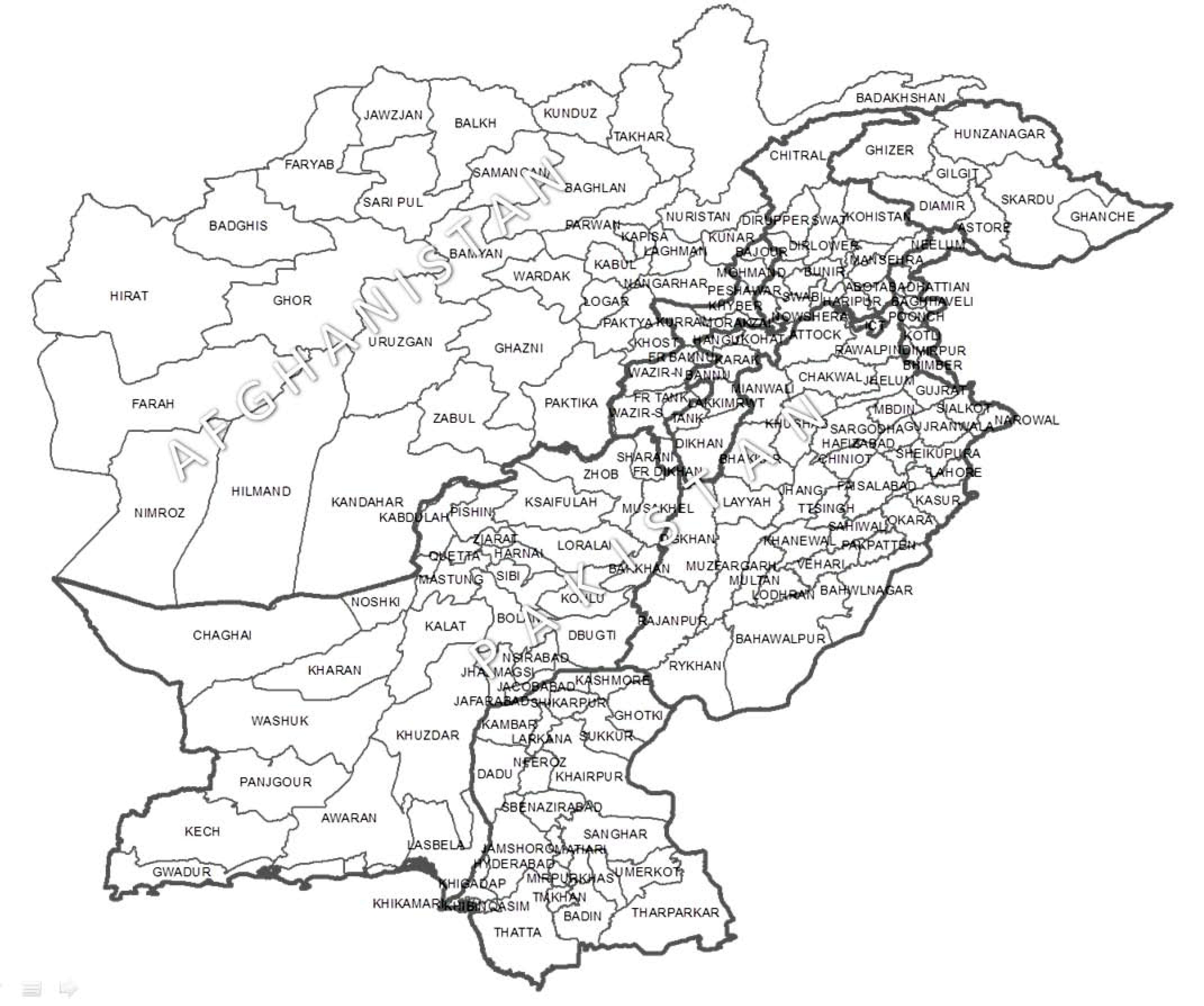

Supplement: Figure S1 — Identifiers of Key provinces and districts in Pakistan and Afghanistan. (TIF) [file pone.0107697.s001.tif]
